# The Influence of Freeze–Thaw Process on the Dynamic Changes in Body Weight and Metal in Groundwater of Seasonal Frozen Lakes: Experimental Study and Model Simulation

**DOI:** 10.3390/toxics13040288

**Published:** 2025-04-09

**Authors:** Hui Zhang, Shengnan Zhao, Xiaohong Shi, Jinda Zhang, Zhimou Cui, Jingyi Wang

**Affiliations:** 1 Water Conservancy and Civil Engineering College, Inner Mongolia Agricultural University, Hohhot 010018, China; 1758065306@emails.imau.edu.cn (H.Z.); imaushixiaohong@163.com (X.S.); 1105872218@emails.imau.edu.cn (J.Z.); hbweczm@163.com (Z.C.); wangjingyi777@emails.imau.edu.cn (J.W.); 2 State Key Laboratory of Water Engineering Ecology and Environment in Arid Area, Inner Mongolia Agricultural University, Hohhot 010018, China; 3 State Gauge and Research Station of Wetland Ecosystem Wuliangsuhai Lake, Inner Mongolia, Bayan Nur 014404, China; 4 Daihai Wetland Hydrology and Ecological Environment Field Scientific Observation and Research Station of Inner Mongolia Autonomous Region, Ulanqab 012000, China

**Keywords:** cold and arid regions, frozen lakes, heavy metals, numerical models

## Abstract

To investigate the changes in heavy metal content in the sub glacial water during the freezing and thawing process of seasonally frozen lakes, the Wuliangsuhai Lake in northern China was taken as the research object. The ice thickness, water depth, and heavy metal content at different depths of the lake were measured during the freezing and thawing periods. Based on a large amount of measured lake heavy metal data, MATLAB 2022b software is used to model data fitting and optimization identification, and wavelet analysis and 24 h sliding average method are used for verification analysis to describe the variation process of heavy metal concentration in ice water with depth and time. The results show that during the freezing and thawing periods of lakes, the water level is constantly changing, but the heavy metal content in the water below the ice follows the same distribution with water depth. During the freezing process, the heavy metal content in the water increases with the increase in ice thickness. A new numerical model describing the spatiotemporal distribution of heavy metals under the ice during the freezing period of the lake was obtained through calculation. The overall trend of the simulated contour lines is consistent with the measured values and has a small error. This study provides a reference for predicting the changes in heavy metal content under the ice cover during the freezing period in cold and arid regions. The model can be used to simulate the content values of heavy metals at different depths and times.

## 1. Introduction

Heavy metals are global environmental pollutants that easily accumulate in organisms and can combine with organic matter to form metal complexes. Furthermore, it accumulates in the food chain and affects human health [1]. In particular, some of the major heavy metal pollutants are arsenic (As), cadmium (Cd), chromium (Cr), copper (Cu), iron (Fe), mercury (Hg), manganese (Mn), lead (Pb), and zinc (Zn). They are widespread in water, soil, and air and can be characterized by their persistence, inconspicuousness, non-biodegradability, and high bioaccumulation [2]. Heavy metals in the environment can enter the human body through the food chain, thus threatening human health. Specifically, although Cu and Zn are non-toxic and essential trace elements for the human body, their excessive intake can have adverse effects on human health [3,4]; Cd exposure primarily occurs through the respiratory and gastrointestinal tracts, leading to various cancers [5,6]; Pb can cause a decrease in intelligence by damaging the central nervous system; Hg not only increases the risk of cardiovascular diseases in adults but also interferes with cognitive development in children. Heavy metal elements can spread and accumulate in the environment, entering lakes through transportation pathways such as sewage discharge and atmospheric deposition, causing serious water pollution, damage to biodiversity, and degradation of ecological functions in lake areas. Therefore, it is necessary to strengthen research on heavy metal pollution, which has become a focus of attention for relevant governments and environmental departments.

Frozen lakes in cold and arid regions account for approximately 40% of the total global lake area [7]. In China, frozen lakes in cold and arid regions account for approximately 2/3 of the total lake area in the country. The characteristic of frozen lakes is that the freezing period can reach up to 7 months, the thickest ice layer can reach up to 1.3 m, and dynamic changes in water level with ice thickness [8,9,10]. Specifically, with the onset of winter, the temperature gradually decreases, and the ice layer begins to thicken. More water under the ice has formed into ice, causing the volume of the lake water to change and the water level to gradually decrease. As the ice thickness increases, the temperature gradually rises, and the ice layer begins to melt; consequently, the pre-compression volume of the water body is restored, leading to an increase in water level. Given these conditions, samples of the upper, middle, and lower water layers need to be collected at different depths during different periods. Therefore, the content of pollutants in the samples can be expected to change with the duration of ice cover. During the freezing of lake water, as the ice thickness increases, pollutants in the original water body are deposited below the ice, resulting in the pollutants being concentrated in the upper water body. During the melting stage of lake ice, the pollutants released first reach the upper water body, leading to a higher concentration of pollutants therein. At the same time, pollutants in the upper water body gradually diffuse downward, and their content gradually decreases with depth. Nevertheless, at the sediment–water interface, pollutants in the sediment are released through the exchange of interstitial water with the overlying water, resulting in the concentration of pollutants in the lower water. Although heavy metals may sometimes precipitate and be trapped in sediments, reducing their concentration in water, during the ice-freezing period, the formation of ice sheets leads to an anaerobic/anoxic environment in the water. The reduction process significantly affects the interface processes of heavy metal adsorption and desorption. Heavy metals adsorbed on mineral surfaces in sediments are easily released, causing secondary pollution of heavy metals [11]. Previous studies have shown that four heavy metals, Cu, Pb, Cr, and Zn, are released from sediments to overlying water at different rates. The corresponding release amounts of the four heavy metals are Zn > Cu > Cr > Pb, with Zn release ranging from 2.80 to 10.10 ug/kg, Cu release ranging from 1.7 to 6.0 ug/kg, Cr release ranging from 0.35 to 1.58 ug/kg, and Pb release ranging from 0.05 to 0.16 ug/kg [12]. Moreover, pollutants in the lower water also diffuse upwards. The overall distribution reflects a dynamic diffusion process of pollutant content with changes in water depth and time. In recent years, most research on heavy metal pollution in lakes at home and abroad has focused on lakes without ice cover [13,14,15,16,17,18], and there is relatively little literature on heavy metal pollution in lakes with ice cover. Wang [19] used a combination of field monitoring and indoor simulation experiments to study the distribution, migration, and transformation of heavy metals in multiple media during the ice-sealing period in Wuliangsuhai. Liu [20] used the QWASI environmental model to study the migration patterns of heavy metals in the multiphase ice-water-sediment environmental media in Wuliangsuhai. Sun [21] introduced the first principles of quantum mechanics and established a microscopic model of heavy metals in ice and water media, exploring the migration mechanism of heavy metals in the lake from the perspective of binding energy. Drbal et al. [22] measured the concentrations of heavy metals in surface water, glacier ice, and plant and animal materials in three areas of Spitsbergen Island in the Svalbard archipelago. Zaborska et al. [23] studied the spatiotemporal variations of heavy metal distribution in Arctic fjords. Poshtegal and Mirbagheri [24] created a one-dimensional qualitative model for the phase transfer of heavy metals dissolved in rivers. However, existing studies mainly focus on the multi-media migration of heavy metals in frozen lakes, and there is no relevant research on the spatiotemporal variation of heavy metal content under the ice in frozen lakes with water depth and freezing duration.

This study analyzed on-site observation data of heavy metal content under the ice in Wuliangsuhai Lake in a typical cold and arid area in northern China in winter from January to April. Vertical distribution profiles of heavy metal content at different depths of the water under the ice at different times were drawn, and changes in the vertical distribution of heavy metal content in the profiles at each time were observed. A C-type distribution model similar to a binomial index was established to describe the spatiotemporal changes in the vertical distribution of heavy metal content under the ice during the freezing period. Numerical simulation showed good fitting with the measured data of heavy metal content. This numerical model can well reflect the spatiotemporal characteristics of heavy metal content under the ice during the freezing period, which depends on the depth of the water and the duration of freezing. It can provide a reference for numerical simulation research on issues of heavy metal pollution in lakes during the freezing period. The model can also be promoted and applied to water environments in other cold and arid regions.

## 2. Materials and Methods

### 2.1. Overview of the Study Area

**Figure 1:**
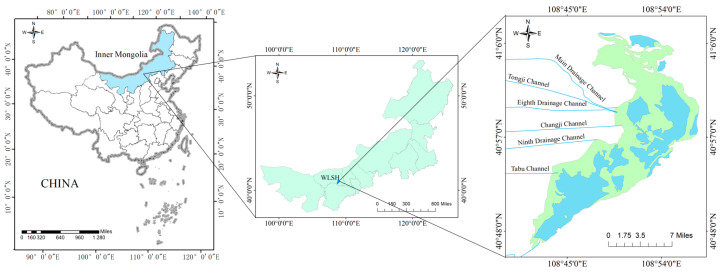
Geographical location of Wuliangsuhai. The green color in the right image represents the reed area, and the blue color represents the clear water area.

Wuliangsuhai (40°36′–41°03′ N, 108°43′–108°57′ E) is located in Urad Front Banner, Inner Mongolia Autonomous Region (Figure 1). It is the largest freshwater lake in the Yellow River Basin, with a total area of 293 km^2^, of which the open water area accounts for about one-third. The water depth is 0.5–2.5 m, and the seabed is essentially flat, with a height difference of approximately 1 m. The annual average temperature is 5.6–7.4 °C, the average precipitation in the basin is 224.2 mm, and the annual water storage capacity is 300 million m^3^ [25]. It is a typical irrigation lake, and its main source of water supply is agricultural water withdrawal from Hetao Irrigation District, accounting for about 80% of the total water replenishment, followed by industrial wastewater, domestic sewage, and floodwater from the surrounding mountains [26]. The lake begins to freeze around early November each year and begins to melt in early April of the following year, with a freezing period of up to 5 months [27]. As the overall water depth is shallow, the ice thickness of the lake generally exceeds one-third of the water depth.

### 2.2. Sample Collection and Measurement

#### 2.2.1. Sampling Method

**Figure 2:**
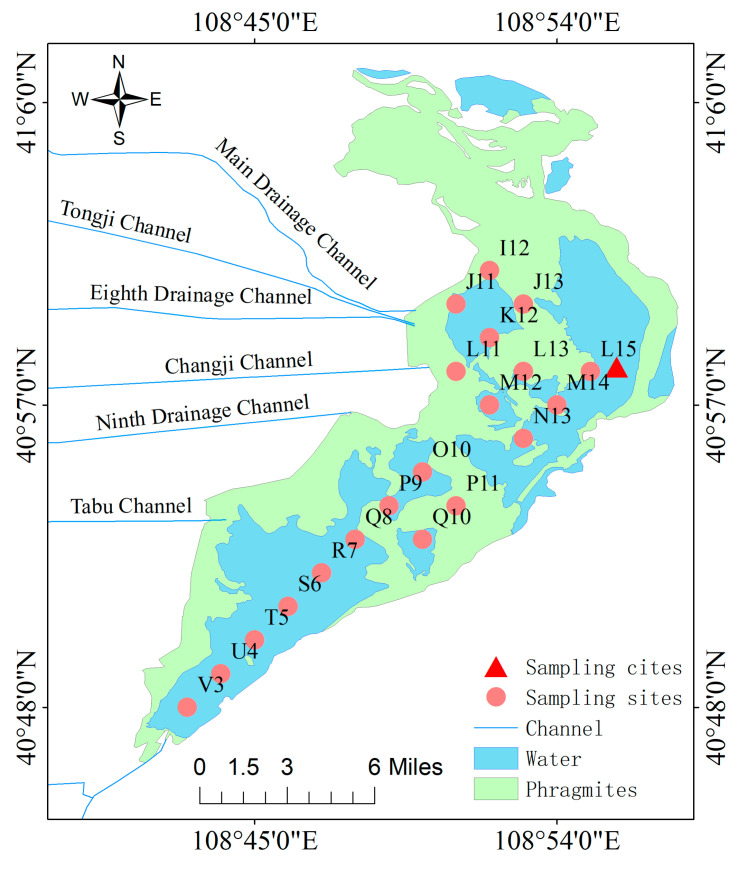
Map of sampling sites in Wuliangsuhai.

**Figure 3:**
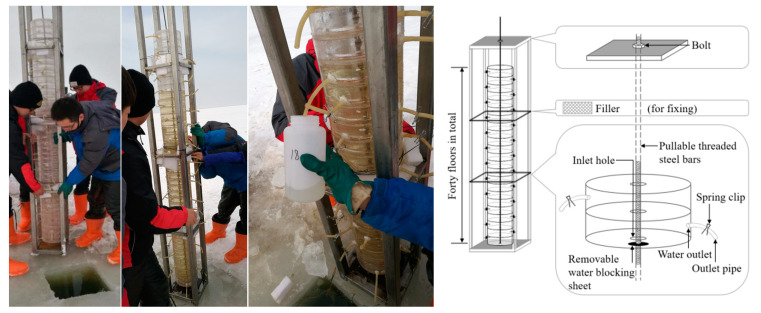
Equipment photos and schematic diagram of stratified water intake.

According to the measurement results of ice and water samples from 21 monitoring points in the entire lake in previous years, the average ice thickness of the lake is 0.64 m, the average water depth is 1.62 m, and the content of metallic Hg in the ice water below point L15 is 1.2025 μg/L [9], exceeding the Class V standard for surface water (1.0 μg/L) and the national fishery water standard (0.5 μg/L). Based on the analysis of data from previous years, the triangle in Figure 2 was selected as the sampling point. At this point, water environmental parameters such as water depth, mud thickness, and ice thickness have reached the average level, and heavy metal pollution is relatively severe. In order to balance the safety of the experimental environment and the integrity of the samples, the sampling time is set from the end of December to mid-January each year. At this time, the Wuliangsuhai enters a completely frozen stage, and winter sample collection and on-site monitoring work continue until the lake begins to melt and the lake ice cannot support on-site sampling work. The first sampling time in the winter of 2018 was on January 4, and the subsequent sampling times were on average every 10 days, including January 17, January 31, February 5, February 15, February 22, March 4, and March 11 as the last sampling, covering 67 days in 2018. The samples were collected every 5 cm up to a depth of 150 cm for a total of 30 layers. Using a GPS column sampler for positioning, a 50 cm × 50 cm square border was drawn at the sampling point. Before sampling, the ice body was cut off along the edges of the square using an electric saw and then removed. After taking the ice body, wait for the water body to stabilize, slowly place the homemade layered water collector into the water (Figure 3) so that the lake water gradually fills each layer of water intake device from the bottom inlet of the layered water collector, with a height of 5 cm for each layer of water intake device, until the bottom layer of the water collector is the mud–water interface. Lift the pull-out threaded steel bar up and tighten the bolts on the top plate of the water collector with a wrench. Therefore, the movable water-blocking plate at the bottom of each layer of water intake container closes the inlet of each layer. Finally, take out the entire water sample and the water collector together, open the spring clamp on the side of each layer, and put each layer of water sample into a polyethylene plastic bottle according to the layer number. Finally, collect 30 layers of water samples. Seal the water sample and bring it back to the laboratory.

#### 2.2.2. Measurement Method

The total amount of heavy metals in the water sample was directly measured without filtration. 25 mL of water sample was taken into a conical flask, and 5 mL of high-grade pure nitric acid (65–68%) was added. The mixture was heated on an electric heating plate at a temperature of (95 ± 5) °C and evaporated until the solution reached about 5 mL. Then, 5 mL of concentrated nitric acid was added until no brown smoke was generated. After cooling, 3 mL of hydrogen peroxide was slowly added and heated until no more bubbles were emitted. Evaporation was stopped until about 5 mL. After cooling, the inner wall of the conical flask and the small funnel were rinsed with deionized water at least three times. After transfer, the mixture was made to a constant volume of 25 mL. An inductively coupled plasma mass spectrometer (iCAP RQ, Thermo Fisher Scientific, Waltham, MA, USA) was used to analyze Ni, Pb, Cu, Cd, Cr, and Zn, while As and Hg were measured using an atomic fluorescence spectrophotometer (AFS-10, Jitian, Beijing, China). A total of 5 mL of the filtered sample was taken and placed in a 10 mL colorimetric tube. 1 mL of hydrochloric acid nitric acid solution was added (300 mL of 36–38% high-grade pure hydrochloric acid and 100 mL of 65–68% high-grade pure nitric acid separately, and 400 mL of water was added and mixed). The mixture was then blocked and placed in a boiling water bath for 1 h of heating and digestion. During this period, the sample was shaken 1–2 times, and the lid was opened to release air. After cooling, the sample was diluted with water to the mark and mixed well before being tested. The quality control during the analysis process adopts methods such as parallel experiments, blank experiments, sample spiked recovery, and standard substance recovery, and the method blanks are all below the detection limit. The drugs used in the experiment are all analytical grade or superior grade, and all containers are soaked in 10% nitric acid for 48 h, then rinsed with deionized water before use. All samples were repeated three times with a relative error of less than 10%.

#### 2.2.3. Quality Control

To ensure the reliability of the experimental data and results, the national standard substance was used for verification. Two parallel samples were set for every five samples, and blank samples and standard samples were also set. The recovery rates of each metal element were within the range of 91.6% to 105.2%, and the relative standard deviation of the measurement was less than 10%. The experimental water used is ultrapure water, and all reagents are of high-grade purity [28].

### 2.3. Data Analysis

This article uses ArcGIS 10.8 software to draw maps of China, Inner Mongolia, and Wuliangsuhai. Excel is used for data organization, MATLAB 2022b software is used for data fitting analysis and prediction drawing, and Origin 2018 software is used to draw other charts.

### 2.4. Model Introduction

Wavelet transform is a local transformation of space (time) and frequency, which performs multi-scale refinement analysis on signals through operations such as scaling and translation. Therefore, it can effectively extract information from signals. In practical applications, continuous wavelets must be discretized. When computers implement data processing, binary discretization is used. Discrete wavelet transform is obtained by discretizing the scale and displacement of continuous wavelet transform to the power of 2, so it is also known as binary wavelet transform.

Moving average is a type of time series filter, which smoothes the short-term volatility to reflect the long-term trends and cycles. Moving average filter smoothes data by replacing each data point with the average of the neighboring data points defined within the span. This process is equivalent to low-pass filtering with the response of the smoothing given by the difference equation.

## 3. Results and Discussion

### 3.1. Vertical Stratification Characteristics of Heavy Metal Content in Lake Water During the Freezing Period

**Figure 4:**
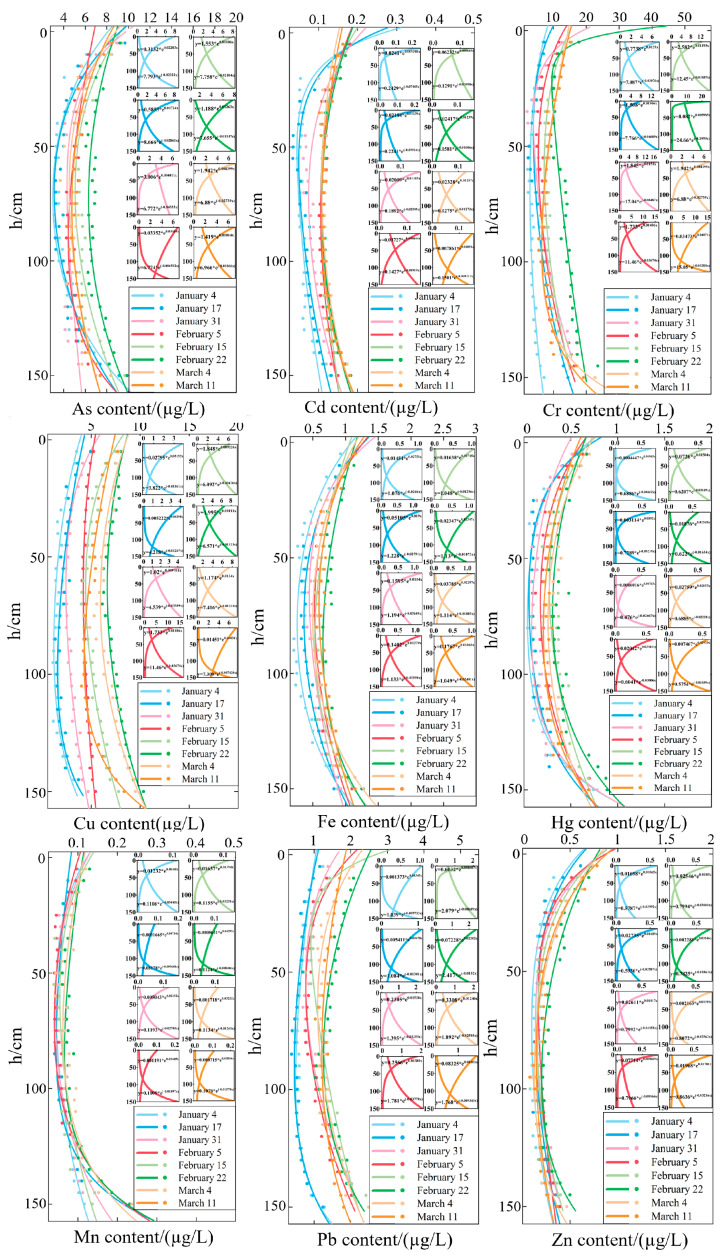
Vertical distribution of heavy metal content at different times. The different colors of the lines represent different dates, and the C-shaped curve on the left is drawn by two small curves on the right.

According to the difference in temperature and dissolved oxygen of the water body, the water temperature from 0 to 15 cm is 18 °C, and the dissolved oxygen is 2.4 mg/L; the water temperature from 15 to 135 cm is 14.6 °C, and the dissolved oxygen is 0.6 mg/L; the water temperature from 15 to 135 cm is 11.5 °C, and the dissolved oxygen is −0.1 mg/L. The maximum temperature difference between the three layers is 2.3 °C, and the maximum dissolved difference is 6.3 mg/L. The water body was divided into three layers, with the top and bottom 15 cm layers being the upper and lower layers and the intermediate being the middle layer. The heavy metal content of each layer of water is shown in Table 1. According to Figure 4, the contents of various heavy metals under the lake ice cover first decreased and then increased with depth. The lowest contents were observed in the middle layer from 50 to 100 cm, and the peak contents were observed in the upper and lower layers. This is because as the ice thickness gradually increases, heavy metals first concentrate in the upper layer. In addition, as metal ions in the water have a larger binding energy, heavy metals in the ice migrate to the upper layer. Therefore, the upper water layer first receives high concentrations of heavy metals and then gradually diffuses downwards. The middle water layer is a transition zone between the upper and lower ones, and therefore, the transfer of water is another factor that influences the lower concentrations of heavy metals. The high concentration of heavy metals in the bottom water is due to the contact between the bottom water and sediments, in which heavy metals in the sediments are released through the exchange between interstitial water and the overlying water.

### 3.2. Characteristics of Metal Stratification in Water Under Different Ice Growth Stages

**Figure 5:**
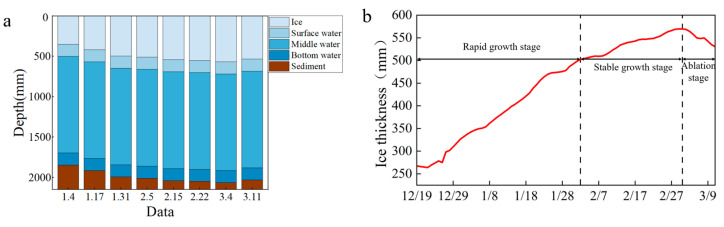
Ice growth in Wuliangsuhai. (**a**) represents that the depth of the upper, middle, and lower water samples obtained each time varies with the thickness of the ice layer; (**b**) represents the three stages of ice thickness changes monitored since December 19.

The variation in ice thickness results in different depths of the upper, middle, and lower water samples obtained each time (Figure 5a), indicating that the content of pollutants in the samples varies with the duration of ice cover. To investigate the effects of time and ice thickness on heavy metal migration, this study monitored the changes in ice thickness from December 19th, as shown in Figure 5b. Based on the changes in the ice cover, the lake ice changes were divided into three stages. The first stage was the rapid growth stage (before February 2), with the ice thickness increasing from 267.41 mm to 492.30 mm. Among them, the ice thickness on December 19 was 267.411 mm, which increased to 346.50 mm on January 4 during the first sampling, 414.48 mm on January 17 during the second sampling, and 492.30 mm on January 31 during the third sampling. The second stage was the stable growth stage (February 3 to March 2), during which the ice thickness increased from 492.30 mm to 569.76 mm. The third stage was the lake ice melting stage (after March 3), during which the ice thickness decreased from 569.76 mm to 542.65 mm.

**Figure 6:**
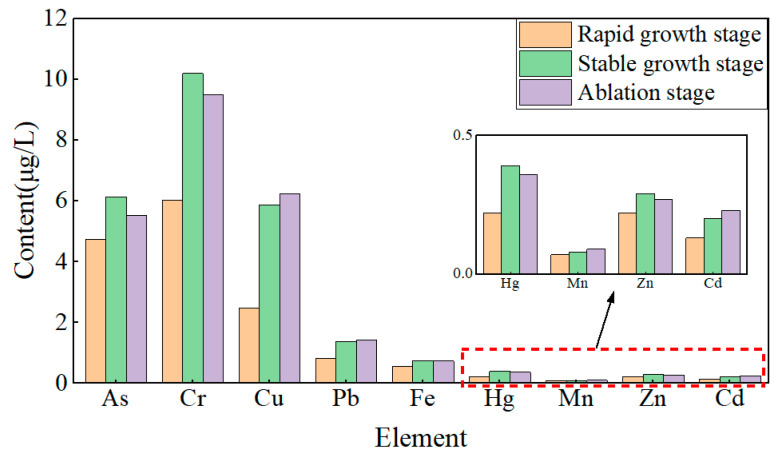
The metal content of water under freezing and thawing process.

During the rapid growth stage of lake ice, the contents of As, Cd, Cr, Cu, Fe, Hg, Mn, Pb, and Zn in the water below the ice were 4.73 μg/L, 0.13 μg/L, 6.01 μg/L, 2.48 μg/L, 0.56 μg/L, 0.22 μg/L, 0.07 μg/L, 0.82 μg/L, and 0.22 μg/L (Figure 6), respectively. During the stable growth stage of lake ice, the growth rate of heavy metal content ranges from 14.29% to 136.69%. During the melting stage of lake ice, the reduction rates of As, Cr, Fe, Hg, and Zn were 10.11%, 6.87%, 1.35%, 7.69%, and 6.90%, respectively. The growth rates of Cd, Cu, Mn, and Pb are 15%, 6.13%, 12.5%, and 4.38%, respectively. Analysis shows that the content of heavy metals As, Cr, Fe, Hg, and Zn increases significantly with increasing ice thickness and slightly decreases with decreasing ice thickness. The content of heavy metals Cd, Cu, Mn, and Pb increases significantly with increasing ice thickness and slightly increases with decreasing ice thickness. In addition, during the three stages of lake ice change, the heavy metal content in the upper and lower layers of water is higher than that in the middle layer, showing a stable C-shaped distribution.

As mid-autumn approaches, with the gradual decrease in temperature, the lake water temperature gradually exceeds the ambient temperature. At this point, the lake begins to release heat into the atmosphere, causing the water temperature to gradually decrease. The temperature of the surface layer of water drops below the freezing point, leading to the formation of ice needles and ice flowers. Over time, these ice crystals gradually rise and gather on the surface of the water, forming a very thin layer of ice. As the negative temperature persists, the ice crystals on the lake surface transform into small, flat, and impurity-free sheet-like structures. These ice crystals usually lie flat on the water surface and gradually grow into star- or tree-like shapes, which are conducive to the release of latent heat. These ice crystals interconnect to form flexible ice, which gradually thickens and eventually forms a hard ice sheet. At the end of November, the temperature in Wuliangsuhai dropped sharply, and the lake rapidly froze. At this stage, the freezing rate exceeded the diffusion rate of pollutants in the lake water, and most pollutants were frozen in the ice. In addition, at this time, the growth morphology of ice crystals is dendritic, with a relatively large surface area, which can capture more impurities. Therefore, during the rapid freezing stage of lakes, most pollutants are frozen in the ice sheet, resulting in higher concentrations of pollutants in the ice sheet compared with the initial ice age and melting period.

In the early stages of freezing, the process of the ice layer gradually thickens over time. Small ice layers first form on the lake’s surface and gradually increase in size. At the same time, a large amount of snowfall accumulates on the surface of the ice sheet, or the lake water flowing out from ice cracks freezes, forming a snow ice sheet. As the freezing rate slows down, columnar ice begins to form, and over time, the lake ice gradually thickens until the end of the freezing period when the ice layer reaches its maximum thickness. Starting from February of the following year, the ice layer of Wuliangsuhai entered a stable growth stage. At this point, the rate of freezing approached the rate of pollutant transport, with the rate of freezing being occasionally lower than the rate of pollutant diffusion. There was sufficient time for pollutants to migrate from the ice to the lower water layer, forming a balance between freezing and migration. At this stage, the ice layer was relatively clean, with lower concentrations of pollutants. Heavy metals have decreased by 1.07–1.38 times compared with the previous stage, with Cu showing the most significant reduction from 1.066 μg/L to 0.788 μg/L, a decrease of 0.278 μg/L. This is because ice has a repulsive effect on pollutants, and as the ice thickness increases, pollutants are discharged into the water body. The ice layer in Wuliangsuhai reached its maximum thickness on March 2, after which it gradually thinned as the temperature increased.

In the late stage of ice growth, with the rise of temperature and the increase in solar radiation, environmental temperature and lake water temperature are higher than the temperature of ice, and the ice sheet begins to absorb heat and gradually melt. At this point, the ice body transforms from an originally hard ice sheet to a loose structure. At this point, the content of heavy metals in the water exhibits different states compared with the previous stage. The content of As, Cr, Fe, Hg, and Zn decreased compared with the previous stage; on the one hand, the melting of ice dilutes the concentration of pollutants in the water beneath the ice. On the other hand, pollutants in the water gradually spread into the loose ice. As the temperature exceeds the freezing point, the ice layer melts from both the surface and the bottom layers. As the snow layer on the ice melts and disappears, the lake water beneath the ice receives direct solar radiation, causing a rapid rise in water temperature. After the complete melting of ice, the flow of water accelerates, resulting in the complete mixing of water and faster diffusion of pollutants. However, the content of Cd, Cu, Mn, and Pb increased compared with the previous stage, which may be due to the presence of external inputs (industrial and agricultural activities, domestic sewage) after the water body completely dissolved, leading to an increase in their content. In addition, the impact of atmospheric deposition may also be one of the reasons for the increase in content.

### 3.3. Model Simulation of Dynamic Changes in Weight and Metal of Water Under Icing Process

#### 3.3.1. Simulation Model Construction

In terms of the vertical spatial distribution, the contents of heavy metals in Wuliangsuhai under the ice cover at different times revealed a C-shaped distribution characterized by an initial decrease, followed by an increase with increasing depth. The vertical distribution profile of heavy metals under the ice cover at different times can be expressed by a C-shaped function that depends on the depth of the water body. Therefore, a C-type distribution model (binomial index model) that depends on the depth of the water body is selected to describe the spatiotemporal distribution of heavy metals under the ice cover:

(1) $$HM\left(h\right)=a\left(t\right)\times e^{b\left(t\right)\times h}+c\left(t\right)\times e^{d\left(t\right)\times h}$$

In the formula, $HM\left(h\right)$ is the heavy metal content (μg/L) at depth h (cm); $a\left(t\right)$, $b\left(t\right)$, $c\left(t\right)$, and $d\left(t\right)$ are fitting parameters. This model is obtained by adding two exponential equations, where $a\left(t\right)$ and $c\left(t\right)$ represent the heavy metal content of the first and second curves at a water depth of h = 0 m, respectively; $b\left(t\right)$ represents the slope of the first heavy metal content distribution curve at a water depth of h = 0 m divided by $a\left(t\right)$; $d\left(t\right)$ represents the slope of the second heavy metal content distribution curve at a water depth of h = 0 m divided by $c\left(t\right)$.

The four parameters $a\left(t\right)$, $b\left(t\right)$, $c\left(t\right)$, and $d\left(t\right)$ in the model could be identified and fitted using mathematical methods based on on-site observation data of different heavy metal contents in the water at different times. Therefore, the model can simulate the heavy metal content in the water during the freezing period of the lake. In order to obtain more accurate feature parameters, observation data from January 4 to March 11 were used to optimize and identify $a\left(t\right)$, $b\left(t\right)$, $c\left(t\right)$, and $d\left(t\right)$ using least squares programming. The corresponding feature parameter values for each time point were obtained. The four sets of data were fitted and analyzed using MATLAB 2022b software, and the results showed that the optimal fitting formulas for the four feature parameters depend on freezing time t and have the same form:

(2) $$Y\left(t\right)=\alpha t+\beta$$

**Figure 7:**
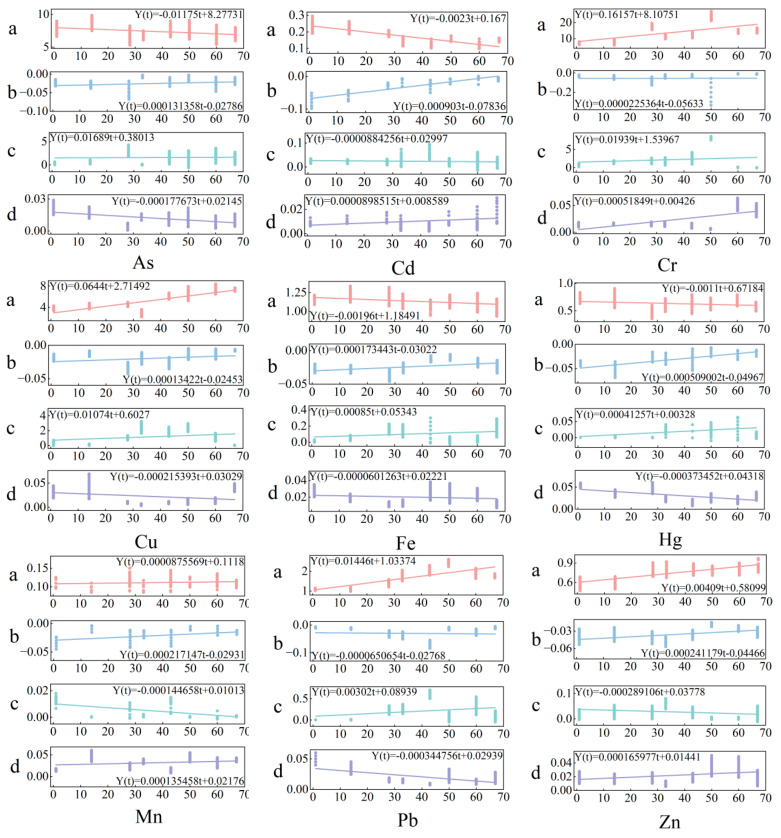
Fitting results of characteristic parameters.

In the formula, $Y\left(t\right)$ represents the characteristic parameters $a\left(t\right)$, $b\left(t\right)$, $c\left(t\right)$, and $d\left(t\right)$, where t is the number of days since the freezing date of the lake surface in the current year, and α and β are coefficient parameters. The fitting results of the coefficient parameters are shown in Figure 7. By using the characteristic parameter equation and coefficient parameters, a graph of four characteristic parameters from January 4 to March 11 could be drawn, as shown in Figure 6. Based on the physical changes in the lake water and the trend of parameter changes, the curve exhibits a similar variation pattern, reflecting the variation pattern of heavy metal content in lake water.

**Figure 8:**
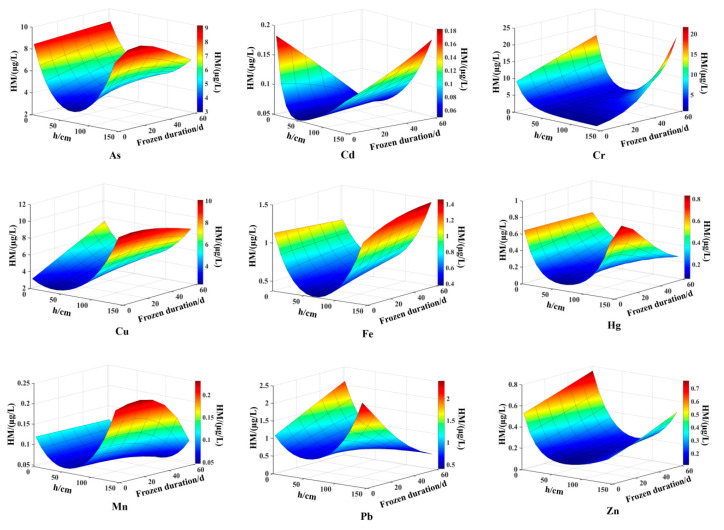
Surface graph of heavy metal content.

The spatiotemporal distribution of heavy metal content in lake water during the freezing period can be simulated and calculated using models and characteristic parameter equations. The distribution of heavy metal content under the lake ice from January 4 to March 11 was analyzed, and a surface graph of heavy metal content was drawn, as shown in the Figure 8. As Wuliangsuhai is a shallow lake located in a cold and arid grassland of northern China, the lake water receives significant solar radiation during the spring months, especially during the melting of ice and snow in April. Kirillin et al. [29] found that establishing a numerical model for lakes during the freezing period requires an understanding of the growth and decline, structure, mechanical and optical properties of seasonal ice-covered lakes, cycles and mixing processes beneath the ice, and a full understanding of physical and chemical reactions in lakes. Therefore, the model and characteristic parameter equation proposed in this study are anticipated to be effectively applied to numerical simulation research on related problems.

#### 3.3.2. Validation of Simulation Models

**Figure 9:**
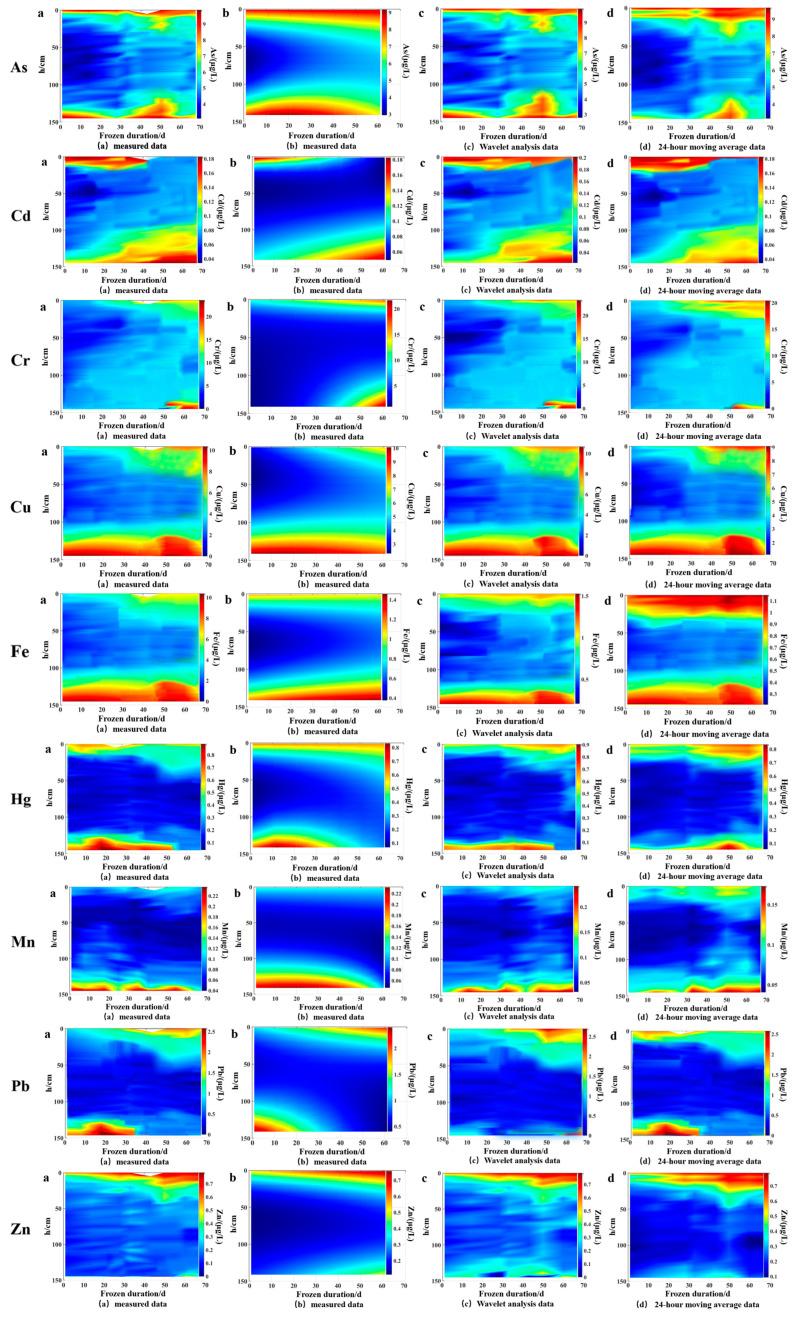
Contour lines of measured and simulated values of heavy metal content. a is the measured content value of each heavy metal, b is the predicted value using the C-type distribution model established in this study, c is the predicted value using wavelet analysis, and d is the predicted value using 24 h moving average method.

Verifying the model with measured values is a crucial step in scientific research aimed at evaluating the degree of fit between the established theoretical model and actual data. When the predicted results of the model match the measured values well, it not only verifies the accuracy of the model but also enhances its credibility in explaining phenomena and predicting future trends. This study plotted contour lines of measured values of various heavy metals with water depth and ice cover duration, as shown in Figure 9a. The contour lines of the model and feature parameter equation simulation calculation are shown in Figure 9b; the contour lines of heavy metal content using the wavelet analysis method are shown in Figure 9c; the contour lines of heavy metal content using the moving average method are shown in Figure 9d. It can be observed that there are many jumping points in the heavy metal content in the wavelet analysis method [30] and the moving average method [31], resulting in uneven contour lines and large errors (4.61~21.98%). This is because the shape and scale of the wavelet function are fixed and cannot adapt to sensitive data changes. In this study, the Cd, Hg, Mn, and Zn contents were small, the amplitude of change was small, and the wavelet analysis method could not make accurate predictions for them; the 24 h sliding average method weights the average of the data and moves it period by period at a certain interval length, which can only predict short-term trends and is not suitable for the 67-day long-term prediction in this study. The model presented in this article has different reference coefficients for different heavy metals, allowing each heavy metal to have its own prediction path and refining the prediction method. At the same time, the modeling process can adjust the smoothness of the graph, which is smoother than the graphs obtained directly from wavelet analysis and the 24 h slip mean method. Comparing the simulated values of the model with the measured values, the data error is between 0.96% and 4.92% (Table 2), indicating that the estimation model for the spatiotemporal distribution of heavy metal content in the water body during the freezing period is more accurate. This means that the model can capture the overall trend of the data, reflecting the continuity and consistency of the model in describing phenomena, ensuring that the model can be effective and reliable in practical applications and can be further analyzed and predicted more reliably based on the model.

#### 3.3.3. Sources of Heavy Metals

Wuliangsuhai is the only receiving water body and drainage channel for farmland irrigation water in the Hetao Agricultural Irrigation Area. It is the only drainage channel for local farmland water, industrial wastewater, and domestic sewage. About 5 × 10^8^ m^3^ of farmland water, 2 × 10^8^ m^3^ of industrial wastewater, and domestic sewage are mainly discharged into the main drainage ditch, eight drainage ditches, and nine drainage ditches through various branch channels and small drainage ditches each year. Nearly 90% of the sewage is discharged into Wuliangsuhai through the main drainage ditch, and the discharged sewage carries heavy metals such as Pb, Hg, As, Cd, Cr, etc. [32,33]. Therefore, agricultural activities, industrial activities, and domestic sewage are the main sources of heavy metals in Wuliangsuhai. Wuliangsuhai is severely affected by human interference [34], and the domestic sewage discharged by human activities mainly contains cleaning agents, which contain a large amount of heavy metals such as Zn and Cr [35]. Zn has strong homology with Cu and Cr elements, so Zn, Cu, and Cr mainly come from domestic sewage; Cd is often regarded as a signature element of agricultural activities [36]. The crops grown in the Hetao Irrigation Area on the Wuliangsu Sea consume a large amount of phosphorus fertilizer every year [37]. Agricultural irrigation and agricultural products such as phosphorus fertilizers emit a large amount of As [38], resulting in higher As content than other heavy metals. Moreover, the Wuliangsu Sea is a typical irrigation lake that receives agricultural non-point source pollution from the basin. Studies have shown that industrial sulfuric acid, phosphate rock, and various organic matter used in the production of fertilizers contain heavy metal elements such as Cd, Pb, Hg, and As. The heavy metals carried by fertilizer runoff flow into the lake, causing an increase in heavy metal content in the water [39,40]. Therefore, Cd, Pb, Hg, and As mainly come from agricultural activities; Wuliangsuhai is located near the Wulashan mining area, where mining and other industrial activities are frequent. Improper discharge of industrial smelting and wastewater can cause Mn and Fe pollution. Therefore, Mn and Fe mainly come from industrial activities [41,42,43,44].

## 4. Conclusions

In this study, the spatiotemporal variation characteristics of heavy metal content in Wuliangsuhai during winter were analyzed. On-site observation data of heavy metal content under lake ice from January 4 to March 11 were analyzed, which revealed a C-type distribution model of the spatiotemporal variation characteristics of heavy metal content in the water under the lake ice. The relevant parameters of the model were optimized, identified, and calculated, and the contents of heavy metals at different depths were numerically simulated. Finally, the spatiotemporal variation characteristics of heavy metal content were statistically analyzed. From the findings, the following conclusions can be drawn:

(1) The curve of heavy metal content under the ice cover and the field data of heavy metals at various depths reveal that driven by the continuous deposition of heavy metals from the ice body to the upper water body and upward release of heavy metals in the interstitial water of the sediments to the bottom water, the upper and lower layers have higher heavy metal contents than the middle layer. Under the action of a concentration gradient and heavy metal diffusion, heavy metals in the water body exhibit a dynamic process.
(2) During the freezing process, the water body under the ice cover generally becomes a closed system without external interference. The ice continuously deposits heavy metals into the water body, and the content of each heavy metal shows the same change characteristic, increasing with the increase in ice thickness. During the melting stage of lake ice, the surface of the lake comes into contact with the atmosphere, and external influences lead to variations in the changing characteristics of the content of various heavy metals.
(3) The C-type distribution model determined the function of heavy metal content under ice cover as a function of water depth and identified characteristic parameters that can be expressed using the best-fitting formula that depends on the ice cover time t. After verification, it was found that the model fits well with the measured data of heavy metal content and can well reflect the spatiotemporal variation characteristics of heavy metal content under ice cover in lakes during the ice cover period that depends on water depth and ice cover duration. It can provide an important reference for the trend of heavy metal content changes under ice cover in other cold and arid regions during the ice cover period.

## Figures and Tables

**Table 1:** Average heavy metal content in different water layers.

	Content (μg/L)	As	Cd	Cr	Cu	Fe	Hg	Mn	Pb	Zn
Aquifer										
upper layer		5.90	0.12	8.07	4.92	0.75	0.33	0.07	1.21	0.36
middle layer		4.78	0.10	7.18	3.98	0.52	0.23	0.06	0.98	0.18
lower layer		6.01	0.12	11.29	5.30	0.75	0.40	0.11	1.39	0.24

**Table 2:** Error between different prediction methods and measured values.

	As	Cd	Cr	Cu	Fe	Hg	Mn	Pb	Zn
C-type distribution model	3.90%	4.12%	2.07%	4.92%	0.96%	3.33%	1.07%	1.21%	2.75%
wavelet analysis	6.79%	4.43%	12.53%	8.60%	4.61%	9.37%	14.58%	9.41%	6.40%
24 h moving average method	9.13%	9.41%	12.54%	8.11%	10.86%	21.98%	13.66%	9.49%	18.91%

## Data Availability

The dataset used in this study is not publicly available due to a data privacy agreement with Inner Mongolia Agriculture University. However, it can be obtained from the corresponding author upon reasonable request.
